# Supplementary material for: Caregivers Like Me: An Education Intervention for Family Caregivers of Latino Elders at End-of-Life
Source: MedEdPORTAL. 2016 Sep 1;12:10448. doi: 10.15766/mep_2374-8265.10448 (PMC6464451; doi:10.15766/mep_2374-8265.10448)
Supplement: Supplementary file 1 — A. Caregivers Like Me Video.mp4 B. Pretest.doc C. Posttest.doc D. PowerPoint Presentation.ppt E. Caregiver Support Example.doc [file mep-12-10448-s001.zip › _Educational Summary Report_10448.pdf]

MedEdPORTAL®
The Journal of Teaching and Learning Resources

Original Publication

OPEN ACCESS

Caregivers Like Me: An Education Intervention for Family Caregivers of Latino Elders at End-of-Life

Dulce M. Cruz-Oliver, MD*, Kirsten Ellis, Sandra Sanchez-Reilly, MD
*Corresponding author: dcruzoli@slu.edu

Citation: Cruz-Oliver DM, Ellis K, Sanchez-Reilly S. Caregivers Like Me: an education intervention for family caregivers of Latino elders at end-of-life. *MedEdPORTAL*. 2016;12:10448. https://doi.org/10.15766/mep_2374-8265.10448

Copyright: © 2016 Cruz-Oliver et al. This is an open-access article distributed under the terms of the Creative Commons Attribution license.

Abstract

Introduction: End-of-life (EOL) care delivery to Latinos is a well-documented challenge. The majority of caregivers for Latino patients are family relatives, but because Latino caregivers may spend more hours in the caregiving role than other ethnic groups and are less likely to use additional available health care, caregivers can experience an increased burden. This can result in Latino elders being more vulnerable to receiving aggressive care. "Caregivers Like Me" was created as a source for nonprofessional caregivers to improve their knowledge about Latino caregiving of elders at end-of-life (EOL). This resource aims to educate about caregiver stress and to improve attitudes towards the utilization of EOL services. **Methods:** "Caregivers Like Me" is a bilingual education intervention that includes a video soap opera, or telenovela. The video is followed by discussion of hospice, palliative care, and caregiver stress definitions and ends with an explanation of services available for caregivers (i.e., social services, support groups, adult day care, chore workers, home care with or without palliative care, and respite care under hospice). **Results:** "Caregivers Like Me" has been demonstrated to improve Latino family caregivers' openness to receiving professional help while caring for their loved ones. Participants in a multisite cross-sectional pilot study among nonprofessional Latino caregivers (N = 145) reported active learning from the intervention and high satisfaction with the overall educational experience. **Discussion:** This tool provides an education format that is culturally and literacy-sensitive to Latino caregivers and effective in changing their attitude toward EOL care. It may be used by professional caregivers to educate Latino caregivers about EOL care.

Keywords

Palliative Care, Culture, Hispanic Americans, Caregivers, Latino, Terminal Care, Hospices, End-of-Life Care, Palliative Medicine, Family Caregivers, Palliative, Early Intervention (Education)

Appendices

A. Caregivers Like Me Video .mp4
B. Pretest.doc
C. Posttest.doc
D. PowerPoint Presentation .ppt
E. Caregiver Support Example .doc

All appendices are peer reviewed as integral parts of the Original Publication.

Educational Objectives

At the end of this module, the participant will be able to:

1. Understand the effect of cultural background on attitudes toward end-of-life care.
2. Identify when additional professional assistance in care is needed because of caregiver stress.

Introduction

End-of-life (EOL) care delivery to Latinos is a well-documented challenge.[1,2] The majority of caregivers for Latino patients are family relatives: *la familia*. Studies have shown that Latino caregivers may spend more hours in the caregiving role than other ethnic groups[3] and are less likely to use available health care services other than their own families[4,5] because they believe family members should provide the care.[6] This lack of health care access can expose caregivers to an increased burden and therefore make Latino elders more vulnerable to receiving aggressive care. Education along with interventions centered on culturally related values may be needed to increase hospice use among Latinos.[7] One study has shown that approaching Latino elders using a novela or telenovela may be a good method to communicate and educate them about health issues.[7] Here, we describe the "Caregivers Like Me" telenovela. Our resource adds to the literature an education intervention that uses the telenovela format to inform about EOL issues and that may even supplement multicomponent interventions[8,9] for Latino family caregivers.

Methods

Development and implementation of this resource have been described elsewhere,[10] but in brief, we started with a literature review[11] to explore Latino elders' EOL care. This demonstrated that Latino elders face EOL decisions with family support and are receptive towards hospice if educated. Subsequently, we performed a needs assessment to study the gaps that exist in EOL care delivery.[11] The needs assessment included focus groups with 45 hospice staff in Texas and Missouri and an experiential journal based on observations in hospice care in Puerto Rico. Observations demonstrated extreme caregiver stress while deciding on nursing home placement of a patient. One solution suggested by focus group participants was to use media to promote EOL education and, specifically, to use a popular TV show format known as telenovelas or soap operas. Telenovelas are usually dramatic stories that aim to transmit sociocultural messages. They are part of the Latino culture and very popular for sharing among families and discussing crucial topics.[12,13]

The needs assessment results were used as a basis to develop an EOL education intervention for Latino caregivers. In particular, we decided to develop a bilingual (Spanish and English) education intervention for Latino caregivers that included a video soap opera (telenovela), "Caregivers Like Me" (Appendix A). The video contains three vignettes, all of them developed at the house of the caregiver protagonist, Margarita Rodriguez, the daughter of the patient with dementia, Don Sanchez. She is receiving a visit from the Lumina Hospice agency because her primary care physician noticed caregiver stress. The first vignette shows how the nurse, Sofia, interviews Margarita and establishes an initial relationship with her. The second vignette shows Dr. Pascual evaluating Margarita for caregiver burden; Sofia joins them, and at the end, opposed to the idea that Margarita has about hospice care, Sofia and Dr. Pascual introduce and offer palliative care services to Margarita. The third vignette starts with the nurse aide interacting with Don Sanchez and Margarita 2 years later after palliative services were started; then, she gives Don Sanchez a bath while Sofia speaks with Margarita and introduces hospice with respite care.

For evaluation of the intervention, a pre- and posttest were created using Kirkpatrick's levels[14] and validated through field testing. The pretest (Appendix B) was designed to capture demographic characteristics of the audience, including age (years), gender (male/female), education (years), patient diagnosis, and Patient Health Questionnaire-2 (range: 0-6; interpretation: higher than 3 is positive for depression)[15] and short Zarit Caregiver Burden Scale (range: 0-16; interpretation: more than 8 is suggestive of caregiver burden) scores,[16,17] in the first eight questions of the pretest, while the last two questions measure pretest attitudes. The short Zarit Caregiver Burden Scale has been validated[16,17] as a screening tool to detect caregiver burden. It consists of four questions about time spent with oneself, time spent with the relative, and feelings of strain or uncertainty about the relative (see pretest question 8). The answers are rated on a Likert scale from 0 (*never*) to 4 (*nearly always*). A score of more than 8 suggests caregiver stress. The posttest (Appendix C) measures changes in attitude (questions 1 & 2), knowledge (questions 4 & 5), and satisfaction (question 3) with experience. Details of telenovela development (e.g., focus group results) will be published elsewhere; following is a detailed description of implementation and resources needed.

Prior to implementation, the facilitator should explore resources for Latino family caregiver support in the area where the intervention will be offered and create a list with contact information for the services. The educational intervention starts with the participant completing the pretest.

The facilitator introduces the topic as follows:

- "Before we start, I would like to give you a few minutes to jot down on the back of your pretest page: What do you expect to learn today?" Provide 30 seconds to 1 minute of silence.
- "Now, I would like you to share your answer with a neighbor." Provide 1 minute.
- "Would anybody like to share your expectations?" The facilitator may comment on some of the expectations, validating them and with the intention to explore their previous knowledge.
- Close with the following statement: "In the next hour, we are going to share with you a video and some information about health care services that may be useful in helping you care for your loved

one. We would like your feedback on what is helpful or not in this meeting. At the end, a space will be provided for questions and more comments."

The facilitator mentions the objectives and key points of the video as follows:

- "At the end of this talk, you will be able to understand the effect of your own culture on attitudes while caring for your loved one, and identify when additional assistance in care is needed because of burden. The main message from the video you will watch are three principles: When making decisions for our loved ones . . .
    - "There is not one solution but a case-by-case approach."
    - "It requires an awareness of the beliefs and values of the caregiver as well as the ones of the patient."
    - "Openness to education and support of professional caregivers."

Participants watch the "Caregivers Like Me" video in the preferred language. After the telenovela, there is a PowerPoint presentation (Appendix D) explaining concepts from the video. Alternatively, the facilitator may choose to use the PowerPoint slides and explain them as detailed below.

- Slide 1: An important topic mentioned in the video is caregiver burden, which stands for the demands (due to a patient's behavior or physical needs) a family member has while caring for the patient. This can be tested for by the physician, using the Zarit scale or just asking the caregiver about his or her mood, level of stress, and sleeping pattern. If any of these affect the caregiver's function, it suggests burden.
- Slide 2: Very often, caregivers self-abandon their health and nutrition and do not exercise, leading to poor self-reported health statuses. Physical burnout due to stress may lead to mental burnout with depression and anxiety. Both things together put caregivers at risk of developing heart disease. Frequently, especially in patients with dementia, when the caregiver suffers from caregiver stress, the family member ends up at the hospital or admitted to a nursing home. The video shows how the early intervention of professional caregivers can delay that admission. This does not necessarily work in every case, but it is a suggestion to take into account.
- Slide 3: What services are out there for caregivers of patients with chronic or terminal illness?
    - *Social service* helps with information about insurance and mental health assistance such as support groups and counseling. For example, in the video, Margarita receives counseling from the nurse Sofia and might even benefit from contacting the Alzheimer Association for a support group. Also, at the end, Sofia helps Margarita look for more help from her own family in caring for Don Sanchez, resulting in one of her brothers moving from Nicaragua to live with her and Don Sanchez.
    - *Adult day care* is a nonresidential facility for supervised care of older adults, providing activities such as meals or socialization a few times per week for some hours. This is especially useful for caregivers who need time to do grocery shopping, visit the bank, or go to work.
    - *Home care* is a service for homebound patients that provides nursing, physical therapy, and social assistance at home, depending on the needs of the patient. For example, in the case of Margarita, she receives nursing assistance for bathing and medical care at home with the goal of providing palliative care to Don Sanchez.
    - *The Program of All-Inclusive Care for the Elderly (PACE)* provides comprehensive long-term services and support to Medicaid and Medicare enrollees in the USA. An interdisciplinary team of health professionals provides individuals with coordinated care. For most participants, the comprehensive service package enables them to receive care at home rather than in a nursing home.
    - *Chore workers* assist with housekeeping, cooking, laundry, and grocery shopping, among other activities. This is a service that is provided through private payment or under insurance coverage (e.g., Medicaid) to certain groups of patients. The service varies depending on the

needs of the caregiver. For example, if a caregiver works, the chore worker may ensure that the patient takes medications and eats breakfast and lunch.
  - *Hospice* is a type of palliative care that provides services at home or at a facility for dying patients. Within hospice, there is a service called *respite care,* which is full 24-hour care provided by the hospice staff (it can also be provided at a nursing home with short stay) while a family member takes 5 days off due to mental or physical burnout. In the case of Margarita, at the end of the video, she receives this care while her brother flies from Nicaragua to join her in caring for Don Sanchez.

The services described in the presentation will vary by region or country. We suggest that the facilitator explore services available in the area where he or she will be providing intervention. A list of local services may be provided by the facilitator at the end of the session (see Appendix E for an example).

The participant then completes the posttest. Alternatively, to explore actual learning, the facilitator may spend a few minutes with the audience discussing question 5 of the posttest: "What did you learn today?" The facilitator then answers any questions related to the video.

Resources needed include a computer with internet and media access for individual participants, a projector connected to a computer with a CD/DVD reader for group sessions, pens/pencils and hard copies of pre- and posttests, and one facilitator. The facilitator distributes the pretest, provides an introduction to the video, and guides the discussion after the video as described above. Then, the facilitator distributes the posttest and collects both pre- and posttests from each individual. The facilitator should not distribute the posttest before the video presentation. Facilitators should have training in either nursing, social work, or medicine. Alternatively, two facilitators may be used so as to avoid leaving the podium empty and to ensure completion of pre- and posttests.

Results

Participants in a multisite cross-sectional pilot study among nonprofessional Latino caregivers ($N = 145$) reported active learning from the intervention and high satisfaction with the overall educational experience.[10] Caregiver stress self-awareness, as well as willingness to accept professional help, improved significantly ($p < .5$) from pre- to posttest.[10] Preliminary data in another multisite cross-sectional study among professional caregivers ($N = 143$) reported posttest improvement in knowledge of cultural competency skills and cultural differences about EOL attitudes, with a tendency for superior improvement in participants who observed the "Caregivers Like Me" telenovela as compared to watching another caregiver video.

Discussion

EOL health care services use by Latino caregivers may be enhanced through this educational tool. "Caregivers Like Me" may serve to overcome cultural challenges by changing family caregiver attitudes to increased use of community support and by increasing professional caregivers' openness to discussing EOL issues with culturally diverse patients. To increase participation among Latinos, group sessions promoted by community leaders are recommended over online individual observation of the video because of the usefulness of discussion among participants and the interaction with the facilitator about the topics of the video.

The use of multimedia group presentation may be challenging due to technological pitfalls (e.g., internet malfunction or ineffective sound system). Therefore, a site visit with preintervention simulation is highly recommended. The pretest and posttest design is appropriate for testing satisfaction and attitude changes but not for measuring learning. Some posttest knowledge questions were too difficult for some study participants. That is why we changed the questions' format to retrospective questions and in this way avoided pretest knowledge bias on posttest answers.

There are numerous limitations to this study. While Latinos have their language and ethnic heritage in common, it is important to note that within the Latino culture, there is heterogeneity and, thus, what applies

to one subgroup may not apply to others. Therefore, one needs to be careful with generalized population statements (in this case, to all Latinos), and facilitators must provide information pertinent to Latino caregivers of the area where the intervention is offered. Participants may respond to the survey, but their responses may not necessarily reflect their true experience. That is why it is important to perform postintervention assessment via phone call or email survey. We performed a one-question phone survey, which demonstrated that participants would probably or most probably intend to use health care services, including home care, palliative or hospice care, chore workers, or support groups, in the event they cared for a loved one in the future.

"Caregivers Like Me" is a valuable educational module that may be used by health care professionals to inform Latino family caregivers about EOL care and the services available to assist them with taking care of their loved ones. It was designed for Latino family caregivers, and we intend to research the effect of this tool on Latino patient outcomes (e.g., hospital, nursing home, or hospice admission rate). Moreover, it would be interesting to explore the module's effectiveness with other ethnically diverse caregivers and their loved ones.

Dulce M. Cruz-Oliver, MD: Associate Professor of Geriatrics, Saint Louis University School of Medicine; Director of Hospice and Palliative Medicine Fellowship, Saint Louis University School of Medicine

Kirsten Ellis: Web Administrator, Saint Louis University School of Medicine

Sandra Sanchez-Reilly, MD: Associate Professor of Medicine, Department of Geriatrics, University of Texas School of Medicine at San Antonio

Acknowledgments
Video production credits: Bo Wich, director; Rick Carrillo, producer; Robb Long, camera; Miguel Cantu and Hector Hernandez, grips; Allen Green, audio; Kiran Panjwani, makeup.

Special thanks to Deborah Villarreal, MD; Miriam Rodin, MD; Nina Tumosa, PhD; Kathy Leonard; and Story Track Studios, St. Louis, MO.

Disclosures
None to report.

Funding/Support
This work was supported by Health Resources and Services Administration (HRSA) Geriatric Academic Career Award K01HP20479-03-00.

Ethical Approval
This publication contains data obtained from human subjects and received ethical approval.

References

1. Hanchate A, Kronman AC, Young-Xu Y, Ash AS, Emanuel E. Racial and ethnic differences in end-of-life costs: why do minorities cost more than whites? *Arch Intern Med.* 2009;169(5):493-501. http://dx.doi.org/10.1001/archinternmed.2008.616
2. Johnson KS, Kuchibhatala M, Sloane RJ, Tanis D, Galanos AN, Tulsky JA. Ethnic differences in the place of death of elderly hospice enrollees. *J Am Geriatr Soc*. 2005;53(12):2209-2215. http://dx.doi.org/10.1111/j.1532-5415.2005.00502.x
3. Dilworth-Anderson P, Brummett BH, Goodwin P, Williams SW, Williams RB, Siegler IC. Effect of race on cultural justifications for caregiving. *J Gerontol B Psychol Sci Soc Sci* . 2005;60(5):S257-S262. http://dx.doi.org/10.1093/geronb/60.5.S257
4. Friedemann M-L, Buckwalter KC, Newman FL, Mauro AC. Patterns of caregiving of Cuban, other Hispanic, Caribbean black, and white elders in South Florida. *J Cross Cult Gerontol*. 2013;28(2):137-152. http://dx.doi.org/10.1007/s10823-013-9193-6
5. Scharlach AE, Giunta N, Chow JC, Lehning A. Racial and ethnic variations in caregiver service use.*J Aging Health*. 2008;20(3):326-346. http://dx.doi.org/10.1177/0898264308315426
6. Carrion IV, Nedjat-Haiem FR. Caregiving for older Latinos at end of life: perspectives from paid and family (unpaid) caregivers. *Am J Hosp Palliat Care*. 2013;30(2):183-191. http://dx.doi.org/10.1177/1049909112448227
7. Crist JD. Latino elder deficits: community partnership. *Nurs Outlook.* 2010;58(2):e7-e8. http://dx.doi.org/10.1016/j.outlook.2010.02.090

8. Llanque SM, Enriquez M. Interventions for Hispanic caregivers of patients with dementia: a review of the literature. *Am J Alzheimers Dis Other Demen*. 2012;27(1):23-32. http://dx.doi.org/10.1177/1533317512439794
9. Gallagher-Thompson D, Coon DW. Evidence-based psychological treatments for distress in family caregivers of older adults. *Psychol Aging*. 2007;22(1):37-51. http://dx.doi.org/10.1037/0882-7974.22.1.37
10. Cruz-Oliver DM, Malmstrom TK, Fernandez N, Parikh M, Garcia J, Sanchez-Reilly S. Education intervention “Caregivers Like Me” for Latino family caregivers improved attitudes toward professional assistance at end-of-life care. *Am J Hosp Palliat Care*. 2016;33(6):527-536. http://dx.doi.org/10.1177/1049909115584315
11. Cruz-Oliver DM, Talamantes M, Sanchez-Reilly S. What evidence is available on end-of-life (EOL) care and Latino elders? A literature review. *Am J Hosp Palliat Care*. 2014;31(1)87-97. http://dx.doi.org/10.1177/1049909113480841
12. Covarrubias KY, Bautista A, Uribe BA. *Cuéntame en qué se quedó. La telenovela como fenómeno social*. Mexico City, Mexico: Editorial Trillas; 1994.
13. Verón E, Chauvel LE. *Telenovela: Ficción Popular y Mutaciones Culturales*. Barcelona, Spain: Gedisa Editorial; 1997.
14. Aretz HT. Some thoughts about creating healthcare professionals that match what societies need. *Med Teach*. 2011;33(8):608-613. http://dx.doi.org/10.3109/0142159X.2011.590389
15. Kroenke K, Spitzer RL, Williams JB. The Patient Health Questionnaire-2: validity of a two-item depression screener. *Med Care*. 2003;41(11):1284-1292. http://dx.doi.org/10.1097/01.MLR.0000093487.78664.3C
16. Martin-Carrasco M, Otermin P, Perez-Camo V, et al. EDUCA study: psychometric properties of the Spanish version of the Zarit Caregiver Burden Scale. *Aging Ment Health*. 2010;14(6):705-711. http://dx.doi.org/10.1080/13607860903586094
17. Regueiro Martinez AA, Perez-Vazquez A, Gomara Villabona SM, Ferreiro Cruz MC. Escala de Zarit reducida para la sobrecarga del cuidador en atención primaria [Short Zarit interview on burden of care for caregivers in primary care]. *Aten Primaria*. 2007;39(4):185-188. http://dx.doi.org/10.1157/13100841

Received: September 2, 2015 | **Accepted:** July 5, 2016 | **Published:** September 1, 2016
