# Supplementary material for: Caregivers Like Me: An Education Intervention for Family Caregivers of Latino Elders at End-of-Life
Source: MedEdPORTAL. 2016 Sep 1;12:10448. doi: 10.15766/mep_2374-8265.10448 (PMC6464451; doi:10.15766/mep_2374-8265.10448)
Supplement: Supplementary file 1 — A. Caregivers Like Me Video.mp4 B. Pretest.doc C. Posttest.doc D. PowerPoint Presentation.ppt E. Caregiver Support Example.doc [file mep-12-10448-s001.zip › E. Caregiver Support Example.doc]

**Algunos servicios en Saint Louis,MO (Services in Saint Louis, MO)**

**Medical Services/Servicios Médicos:**

**Casa de Salud - (314) 977-1250**

-just $25 fee, no other insurance/documents needed

-solamente cobran 25$ por el servicio, ningún otro document o seguro medico requerido

**Grace Hill – (314) 814-8778**

-sliding scale fee, medicare/medicaid, blue cross accepted

-cobran por verificar azucar, aceptan los seguros médicos Medicare/Medicaid & blue cross

**Family Care Health Care: (314) 531-5444**

-provide primary care services with or without insurance

-provee servicios de medicina primaria no importa si tiene o no seguro medico

**Home Care/Servicios a domicilio:**

**St Louis Home Health – (314) 352-7889**

**Active Angels – (314) 524-4200**

**Alexian Brothers PACE program - (314) 771-5800**

-fully covered by Medicare/Medicaid

-cubierta complete bajo el plan medico Medicare/Medicaid

**Palliative Care/Cuidados paliativos y de hospicio:**

**Nurses and Company Hospice: (636) 926-3722, (314) 685-3722, (618) 310-1591**

**BJC Hospice: (314) 872-5050**

**Gentiva Hospice: (314) 574-4645**

**Charitable hospices for uninsured patients:**

**Crossroads Hospice: (314) 801-6960**

**VITAS Innovative Hospice: (314) 682-3400**

**Adult Day Care/Cuidado diurno para adultos:**

-**Sunvalley adult day care center- (314) 868-2232**

-**St. Elizabeth adult day care center- (314) 772-5107**

**Chore Worker/Ama de llaves:**

**St Louis Housing Authority-**	(314) 535-3616

-Must undergo a Needs Assessment with them

-Se require una evaluación inicial antes de recibir los servicios

**Dream Team Community Services – (314) 522-6414**

-Must be referred by social services but is a free service

-Se requiere un referido del trabajador social, pero es un servicio libre de costo
