# Supplementary material for: Caregivers Like Me: An Education Intervention for Family Caregivers of Latino Elders at End-of-Life
Source: MedEdPORTAL. 2016 Sep 1;12:10448. doi: 10.15766/mep_2374-8265.10448 (PMC6464451; doi:10.15766/mep_2374-8265.10448)
Supplement: Supplementary file 1 — A. Caregivers Like Me Video.mp4 B. Pretest.doc C. Posttest.doc D. PowerPoint Presentation.ppt E. Caregiver Support Example.doc [file mep-12-10448-s001.zip › B. Pretest.doc]

***Latino End of Life Initiative***

***Caregivers DESCRIPTIVE & PRETEST***

1. What sex where you assigned at birth? **1. Female	2.Male**
2. How old are you? ________ **years old**
3. What is your ethnicity? **1. White	2. Black	3.Hispanic/Latino	4.Asian	5. Pacific Islander	6. American Indian	7.Other:_________________**
4. How much education have you completed?
1. **Elementary School 2.High School 3.College 4.More than college 5. Other (specify):**_________
5. Select all chronic illness of my relative that I have most recently cared for (circle all that apply):

**1. Dementia 2. Cardiovascular disease (including heart disease, hypertension or stroke) 3.Cancer 4.Other (specify): __________________________**

6. In general, would you say your health is excellent, very good, good, fair or poor? (circle one):

**1. Poor	2. Fair	3.Good	4. Very Good	5. Excellent**

7. Patient health questionnaire (PHQ-2)
   a. Over the last 2 weeks, how often have you been bothered by little interest or pleasure in doing things? Circle one.

**0.Not at all	1. Several days	2. More than half the days	3. Nearly every day**

b. Over the last 2 weeks, how often have you been bothered by feeling down, depressed or hopeless?

**0.Not at all	1. Several days	2. More than half the days	3. Nearly every day**

8. Caregiver burden scale (SHORT ZARIT-4)
   a. Do you feel that because of the time you spend with your relative that you do not have enough time for yourself?

**0.Never	1. Rarely	2. Sometimes	3. Quite frequently	4. Nearly always**

b. Do you feel stressed between caring for your relative and trying to meet other responsibilities (work/family)?

**0.Never	1. Rarely	2. Sometimes	3. Quite frequently	4. Nearly always**

c. Do you feel strained when you are around your relative?

**0.Never	1. Rarely	2. Sometimes	3. Quite frequently	4. Nearly always**

d. Do you feel uncertain about what to do about your relative?

**0.Never	1. Rarely	2. Sometimes	3. Quite frequently	4. Nearly always**

9. Rate if you “strongly disagree, disagree, neither agree nor disagree, agree, or strongly agree” with the following statements:

|  | **Strongly disagree** | **Disagree** | **Neutral** | **Agree** | **Strongly agree** |
| --- | --- | --- | --- | --- | --- |
| a. It will be stressful for me to take care of an elderly relative with terminal illness. | 1 | 2 | 3 | 4 | 5 |
| b. It is essential for the well-being of caregivers to seek assistance from agencies (e.g. home care, hospice, social services, etc.) to help care for their relative with advanced disease. | 1 | 2 | 3 | 4 | 5 |

10. If you are caring for a relative with terminal disease, would you accept help at home from any of the following agencies?

|  | **No** | **Maybe No** | **Neutral** | **Maybe Yes** | **Yes** |
| --- | --- | --- | --- | --- | --- |
| a. Home care: provides nursing, physical therapy and social assistance at home | 1 | 2 | 3 | 4 | 5 |
| b. Hospice: provides nursing and social services at home for dying patients | 1 | 2 | 3 | 4 | 5 |
| c. Social service: helps with insurance application and mental health assistance | 1 | 2 | 3 | 4 | 5 |
| d. Adult day care: non-residential facility for supervised care of elders a few times per week | 1 | 2 | 3 | 4 | 5 |
| e. Chore worker: assist with housekeeping and cooking among other activities | 1 | 2 | 3 | 4 | 5 |
