# Supplementary material for: Caregivers Like Me: An Education Intervention for Family Caregivers of Latino Elders at End-of-Life
Source: MedEdPORTAL. 2016 Sep 1;12:10448. doi: 10.15766/mep_2374-8265.10448 (PMC6464451; doi:10.15766/mep_2374-8265.10448)
Supplement: Supplementary file 1 — A. Caregivers Like Me Video.mp4 B. Pretest.doc C. Posttest.doc D. PowerPoint Presentation.ppt E. Caregiver Support Example.doc [file mep-12-10448-s001.zip › C. Posttest.doc]

***Latino End of Life Initiative***

***Caregivers POSTTEST***

1. As a result of attending this meeting rate if you “strongly disagree, disagree, neither agree nor disagree, agree, or strongly agree” with the following statements:

|  | **Strongly disagree** | **Disagree** | **Neutral** | **Agree** | **Strongly agree** |
| --- | --- | --- | --- | --- | --- |
| a. It will be stressful for me to take care of an elderly relative with terminal illness. | 1 | 2 | 3 | 4 | 5 |
| b. It is essential for the well-being of caregivers to seek assistance from agencies (e.g. home care, hospice, social services, etc.) to help care for their relative with advanced disease. | 1 | 2 | 3 | 4 | 5 |

2. While caring for your loved one with terminal illness, if it is necessary would you request any of the services suggested in the meeting?

|  | **No** | **Maybe No** | **Neutral** | **Maybe Yes** | **Yes** |
| --- | --- | --- | --- | --- | --- |
| a. Home care: provides nursing, physical therapy and social assistance at home | 1 | 2 | 3 | 4 | 5 |
| b. Hospice: provides nursing and social services at home for dying patients | 1 | 2 | 3 | 4 | 5 |
| c. Social service: helps with insurance application and mental health assistance | 1 | 2 | 3 | 4 | 5 |
| d. Adult day care: non-residential facility for supervised care of elders a few times per week | 1 | 2 | 3 | 4 | 5 |
| e. Chore worker: assist with housekeeping and cooking among other activities | 1 | 2 | 3 | 4 | 5 |

3. How satisfied are you with the overall educational experience you received at this seminar:

**1. Very dissatisfied	2. Dissatisfied	3.Unsure	4. Satisfied	5. Very satisfied**

4. Rate your knowledge from “low to high” (1=low to 5=high) on the items below: A) BEFORE you attended this seminar and B) AFTER you attended this seminar.

|  | KNOWLEDGE **BEFORE** SEMINAR | KNOWLEDGE **AFTER** SEMINAR |
| --- | --- | --- |
|  | Low High | Low High |
| a. Knowledge of home health care | 1 2 3 4 5 | 1 2 3 4 5 |
| b. Knowledge of palliative care services (e.g. hospice) | 1 2 3 4 5 | 1 2 3 4 5 |
| c. Knowledge of social services | 1 2 3 4 5 | 1 2 3 4 5 |
| d. Knowledge of adult-day care | 1 2 3 4 5 | 1 2 3 4 5 |
| e. Knowledge of resources available to elders through the local agency for aging (e.g. chore worker) | 1 2 3 4 5 | 1 2 3 4 5 |

5. What did you learn from this module? _______________________________________________________________________________________________________________________________________________________________________________________________________________________________________________________________________________________
